# Silent SARS-CoV-2 Infections, Waning Immunity, Serology Testing, and COVID-19 Vaccination: A Perspective

**DOI:** 10.3389/fimmu.2021.730404

**Published:** 2021-09-21

**Authors:** Madhusudhanan Narasimhan, Lenin Mahimainathan, Jungsik Noh, Alagarraju Muthukumar

**Affiliations:** ^1^Department of Pathology, University of Texas Southwestern Medical Center, Dallas, TX, United States; ^2^Lyda Hill Department of Bioinformatics, University of Texas Southwestern Medical Center, Dallas, TX, United States

**Keywords:** asymptomatic, waning, serology testing, COVID-19, vaccination

## Abstract

Severe acute respiratory syndrome coronavirus 2 (SARS-CoV-2) virus causes a spectrum of clinical manifestations, ranging from asymptomatic to mild, moderate, or severe illness with multi-organ failure and death. Using a new machine learning algorithm developed by us, we have reported a significantly higher number of predicted COVID-19 cases than the documented counts across the world. The sole reliance on confirmed symptomatic cases overlooking the symptomless COVID-19 infections and the dynamics of waning immunity may not provide ‘true’ spectrum of infection proportion, a key element for an effective planning and implementation of protection and prevention strategies. We and others have previously shown that strategic orthogonal testing and leveraging systematic data-driven modeling approach to account for asymptomatics and waning cases may situationally have a compelling role in informing efficient vaccination strategies beyond prevalence reporting. However, currently Centers for Disease Control and Prevention (CDC) does not recommend serological testing either before or after vaccination to assess immune status. Given the 27% occurrence of breakthrough infections in fully vaccinated (FV) group with many being asymptomatics and still a larger fraction of the general mass remaining unvaccinated, the relaxed mask mandate and distancing by CDC can drive resurgence. Thus, we believe it is a key time to focus on asymptomatics (no symptoms) and oligosymptomatics (so mild that the symptoms remain unrecognized) as they can be silent reservoirs to propagate the infection. This perspective thus highlights the need for proactive efforts to reevaluate the current variables/strategies in accounting for symptomless and waning fractions.

## Introduction

This is a perspective chiefly based on the reports that have suggested antibody quantitation could be a prevaccination screening strategy and specifically, a single dose of COVID-19 vaccine may likely suffice for the already SARS-CoV-2 infected cohort (1–5). These studies along with other findings similar to ours, have shown that the serological assessment of nucleocapsid (N)- and spike (S)-specific IgG antibody levels could differentiate vaccine-induced responses from those acquired following SARS-CoV-2 infection (1, 6, 7). In addition, these interesting and elegant studies demonstrated that the first dose (1D) of mRNA vaccine generated similar protective antibody responses in previously SARS-CoV-2 infected healthcare workers to that of a second dose (2D) seen in immunologically naïve patients (1, 2, 4, 5). Further, the modest ACE2 binding inhibition responses of 2D *versus* 1D vaccine doses among COVID-19 recovered individuals reassures that a single dose could govern a protective antibody response in this population (1). This emphasis concerning the potential for single dose vaccination in prior COVID-19 individuals is relevant and timely, given the drastic decrease in new cases reported with that approach in many countries alongside a prolonged antigenic stimulation that has the likelihood of dampening the immune response *via* effector T-cells exhaustion, as has been observed with several other viruses (8). Among the vaccinated, there appears to be some difference in the extent and duration of immune response depending on the number of doses taken, time gap given between the first and second dose, prior infection/disease burden besides the physical, environmental, and general health status’ influences (9). Based on some select studies, **Table 1** enlists some key responses and differences seen after administration of 1D and 2D of COVID-19 vaccines.

**Table 1:** Some key responses and differences seen after 1D and 2D of COVID-19 vaccines.

S.No.	Particulars	1D	2D	Comments	Reference
1.	IgM	Low	High	Low and high was relative to the unvaccinated controls. IgM levels were found to be *increased* **** by 1.7-fold in the 2D-received naïve group (seronegative) with no appreciable change in the prior infected group. This was, however, only transient and during the initial period following vaccination.	(7)
2.	IgG	Low	High	Low and high was assigned based on the relative levels with the pre-vaccine status. Median IgG levels was *increased* by 7.0-fold in sero+ 2D group; 8.6-fold in sero- 2D group; no change in the prior COVID-19+ group; with ~1.8-fold in the overall 2D population that includes sero+, sero-, and prior COVID-19+ subgroups.	(10)
3.	Virus neutralizing potency	Low	High	Low and high was based on the neutralization antibody titers relative to pre-vaccine (1D). The median potency of 2D when adjusted and compared to 1D was *increased* in the range between 2.6 and 26-fold in sero- group; between 1.3 and 1.7-fold in 2D sero+ group.	(10, 11)
4.	Vaccine Efficacy- (VE)			❖ VE* was assessed in terms of onset of COVID infection and a low VE indicates a high infection. VE was found to be 52.4% in the period between 1D and 2D and was *increased* to 92.7% at 2 or more days after 2D. ❖ In a multicenter SIREN study including 23,324 participants from 104 sites (*all in England*), the VE^#^ assessed in terms of new infections observed at ≥21 days after 1D was reduced by 50% at 7 days after 2D. ❖ In a nationwide historical cohort study from *Israel* with 6286 subjects, between ≥14 days after 1D until the receipt of 2D, VE^@^ was found to be 61%, which was increased to 82% from 1 to 6 days after the 2D.	(12) (4) (13)

*VE was derived according to the Clopper–Pearson method using the formula 100× (1−IRR), where IRR is the calculated ratio of confirmed COVID-19 cases per 1000 person-years of follow-up in the active vaccine group to the corresponding illness rate in the placebo group.

^#^VE was deduced from the incidence density of new infections/10,000 person-days (8 following 1D vs 4 after 2D).

^@^VE was deduced from the effectiveness of vaccine against PCR positive SARS-CoV-2 (with or without symptoms).

Along these lines, the potential biases around presumed high proportion of ‘silent’ asymptomatic patients must be duly acknowledged. Also concerning is the nebulously defined asymptomatic testing by Centers for Disease Control and Prevention (CDC) guidelines on one hand (14–17), and its relaxed guidelines on masking and distancing for FV population that can still contract breakthrough infections. Together, these clearly portend the need for careful surveillance/assessment mechanism(s) for the symptomless and distinguishing them from presymptomatic cases. Notably, as of May 25, 2021 CDC’s report, about 27% (2725/10262) vaccine breakthrough infections were asymptomatic, and in those, 29% (289/995) of hospitalizations were related to asymptomatic or unrelated to COVID-19 (18). While some anecdotal evidence raises optimism that asymptomatic-driven transmission of the infection can subtly result in comprehensive immunization of the population towards herd immunity. There are other studies that report only one in five asymptomatic carriers possesses the capacity to seroconvert compared with severe and mild COVID-19 cases during or after hospitalization (19). Thus, it is tenuous whether asymptomatic infections can allow protective immunity. Hence, we believe it is the right time to proactively characterize asymptomatics and oligosymptomatics from such studies that assess and deduce prevalence-based protection and prevention measures. Only then, the challenges surrounding vaccine redirection to hotspots/appropriate groups, mitigation of vaccination inequities, and efforts to enhance the speed, coverage, and impact of vaccination across the globe will be tackled adeptly.

## Asymptomatic Infections and Waning Immunity Remain as an Uncharted Territory in the Understanding of SARS-CoV-2 Infection

**Figure 1:**
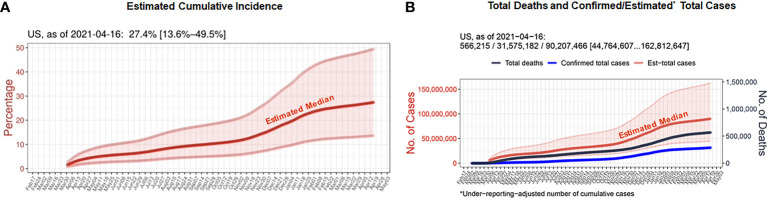
**(A)** Estimates of cumulative incidence rates for the period until April 16, 2021, for the 50 U.S. states. **(B)** Seven-day rolling-averaged counts of daily confirmed total cases and deaths until April 16, 2021, for the U.S. For this computational study, the COVID Tracking project provided the U.S. dataset (20) and the Center for Systems Science and Engineering (CSSE) repository at Johns Hopkins University remained as a source of confirmed cases and deaths for countries (21). The actual number of infections across countries and regions were inferred in terms of the Infection-Fatality-Rate (IFR), since it is one of the key epidemiological parameters that afforded us a clue to fill the gap between confirmed and actual infections, under the assumption that the number of undocumented deaths is negligible (14). While the IFR is subjected to fluctuations depending on age structure of population, timeline, the current estimation uses a consensus and previously established estimate of 0.66% IFR that encompass a wide band of uncertainty (0.39%–1.33%, 95%-confidence interval) among all the PCR-confirmed infections including asymptomatic cases (22). It is worth noting that considering the estimate’s large estimation uncertainty, the confidence interval is expected to cover the true IFRs of most countries and U.S. states and our machine-learning-based IFR estimates and current framework of daily counts of ‘actual’ COVID-19 infections were in line with the existing seroprevalence rates in 46 U.S. states (14).

A vast majority of the studies alluding to emphasize the potential of a single dose vaccination strategy in prior infected cohorts has included only the confirmed cases (symptomatic) and assessed the effectiveness of vaccination in terms of quantifying the antibody levels (1–7). However, it is to be noted that a significant percentage of COVID-19 infections were silent/asymptomatic causing many infections to go unreported (14–17). Using a new machine learning algorithm developed by our team (accounting for undocumented infections), we have reported that the total numbers of predicted COVID-19 cases to be significantly higher than reported across the nation and worldwide (14). Represented in the **Figures 1A, B** is an estimated cumulative incidence (~27%) and estimated total current infections (90 million) across the U.S, respectively as of April 16, 2021. This is very critical given the relevant published study’s (1) central theme is that a single dose vaccination could be sufficient for prior infected, for which an accurate estimation of the true size of infected population is pivotal. Note that stringent reliance on confirmed cases only can lead to under-ascertainment of COVID-19 infections (14). Not accounting for the asymptomatic and oligosymptomatic population in the context can mislead the experts in gauging the vulnerability of a community to the virus and confound the subsequent decisions on mitigation strategies. Could strategic surveillance testing using adequate follow-up (serial PCR) and orthogonal immunological assessments (antigen- and antibody- based tests) of people that are likely exposed to confirmed cases (e.g., contact tracing) or are high-risk spreaders (e.g., front-line and congregate facility workforce) be leveraged along with systematic data-driven modeling approach (integrating age, sex, chronic conditions and COVID-19 risk factors, etc.) allow better characterization of infection dynamics (proliferation, clearance, and persistence) in asymptomatic pool and ably guide the planning and optimization of specific actions?

Compounding the asymptomatics, waning immunity, *per se*, may challenge the testing and interpretation with false negative immunoassay results because of decreasing levels of antibodies and higher positive cut-off thresholds set by vendors that were mainly derived from active infection cases. Importantly more the ‘interacting asymptomatics and oligosymptomatics (e.g.: working age)’ socially mix with the non-immunized or immunized (under waning immunity), greater and sustained will be the spread of infection. Thus, a disruption in the timing and intensity of interventional strategies and/or efforts is imminent when the asymptomatic and waning immunity considerations are discounted. While the effectiveness of authorized COVID-19 vaccines is apparent from real-world scenarios and a spate of clinical studies, the emergence of new variants and reports of a worldwide surge of recent vaccine breakthrough infections with the Delta variant (B.1.617.2) of coronavirus in the FV have raised alarms about the waning vaccine immunity (23). In an unreviewed study that evaluated the mRNA vaccines’ longitudinal effectiveness across different states in the U.S. including Minnesota, Wisconsin, Arizona, Florida, and Iowa between January and July 2021, the efficacy of Pfizer vaccine was found to have dropped by nearly two-fold than Moderna (24). An Israel study has also underscored the concerns of rising breakthrough infections with an ebb in vaccine’s efficacy by reporting 2.26 times greater risk of infection in the early Pfizer vaccinees (Jan-Feb, 2021) compared to those vaccinated later (Mar-Apr, 2021) (25). However, so far, the rate of breakthrough infections reported in vaccinated population is modest compared to the soaring new infections in unvaccinated populations. Notably, prior vaccination appears to be strongly reducing the risk of hospitalization and developing severe COVID-19 in non-immunocompromised individuals. Yet unbeknownst, whether the diverging effectiveness of COVID-19 vaccines is owing to inherent differences in potency of vaccines against the Delta variant or their varying durability characteristics, the need and value of an additional dose to refresh the fading immunity in the general population has become a subject of intense scientific debate in the COVID community. However, to translate this idea of additional doses as ‘a’ key to stop the pandemic into reality, a speedy coverage of remaining worldwide population that is yet to receive either 1D or 2D of vaccines coupled with efforts to properly track and understand breakthrough infections in real-time is equally important.

From the laboratory-based analysis, to monitor such circumstances, binding immunoassay format such as anti-nucleocapsid-pan-Immunoglobulin (anti-N-pan-Ig) electro-chemiluminescence assay (ECLIA) that detects late, mature, high affinity antibodies regardless of the subclass with high sensitivity and specificity from <5 days (proportion of infection detected only by PCR) until >15–22 days samples post-symptom when used in serial measurements could come in handy (26). It is notable that CDC recommends serial serological screening and surveillance testing to identify carriers with asymptomatic infections and waning conditions (17). However, feasibility of screening chiefly used in the setting of outbreaks or in high prevalent areas and how it must fit in this context must also be considered along with other potential alternatives. Yet, amidst the ambiguity, very recently (July 27, 2021), the CDC has made an encouraging recommendation by reversing the previous testing exemption granted for FV with no COVID-19-like symptoms even after a close contact with confirmed COVID-19 patient(s) to mandating the testing for FV who still don’t show symptoms after an exposure (27).

## Is There a Case to Consider an ‘Editable Threshold’ of Serology Assays to Reveal Previously Undiagnosed Infections?

The bigger purpose of studies focused on understanding if a single dose vaccination is sufficient for prior-infected subjects (1–5) must primarily involve identification and clustering of the truly infected (prior) from uninfected subjects. Serological evaluation of SARS-CoV-2 antibody profiles is an important tool to assess prior SARS-CoV-2 infection and infection prevention strategies. However, the SARS-CoV-2 antibody levels become low as in mild infections or decline over time owing to waning immunity and thus, using manufacturer-established PC thresholds of N-IgG can underestimate actual case numbers, yielding an incomplete number of true past infections (8). Relevantly, most informative data to improve the identification of individuals either prior-infected and resolved or under waning immunity came from the idea of orthogonal testing and an in-depth optimization of the manufacturer-established positive cut-off (PC) of N-IgG assay without compromising assay specificity (28–30). Under the ‘editable gray-zone’ threshold, the European Union recently also approved refinement of Abbott’s IgG SARS-CoV-2 assay, allowing laboratories to adapt PC carefully and achieve a ‘near-perfect’ quantification of infected subjects (*Personal communication with Abbott*). Notably, a misclassification of prior-infected individual as uninfected can severely impact the health, economic, and social picture, which can complicate actual intent of effective COVID-19 single-dose vaccination strategy. While the study uses antibody testing results as one criterion, broaching on the concept of an in-depth serology testing was circumvented in these studies. If a reliable solution is of ultimate interest, it is critical to ensure an optimal clustering of the ‘target’ population (prior-infected) for 1D vaccination. Pertinently, a careful reflection of data collection on orthogonal testing and alternative data analysis approaches like editable serology cut-off could thus be more germane.

Many reports, including Ebinger’s study, identified a fraction of the naïve individuals following 1D reached the neutralizing threshold titer or beyond (for instance, nearly 8% in the 1D category; Figure 1 from Ref #1). This raises important questions whether these naïve individuals that exhibited a hyper-IgG response following 1D (compared to the prior-infected individuals) were truly naïve, or perhaps asymptomatically infected, or their samples collected at a later period within the 7-21 days (≥14 days, where typical IgG response is highly likely). This can be addressed (i) if those naïve group subjects who had all 3 data points (baseline, 1D and 2D) are plotted longitudinally per individual basis and (ii) by providing distribution of antibody response over time for the 1D and 2D separately. These data are critical in obtaining a clearer picture when defining a cohort for a single dose vaccination. Moreover, vaccine-triggered protective immunity is also known to decay progressively and wane over time, requiring revaccinations. In such situations, studies reflecting the accurate prevalence and persistence of infection (e.g.: accounting for asymptomatics) and immune status/sustainability (e.g.: waning) could be valuable to illuminate the impending patterns of oscillating infection(s)/outbreaks. This, in turn, can help guide and drive an effective periodic immunization program (e.g.: extending immunity duration *via* administering another booster).

## Is It Worth Establishing a Pan Ig: Neutralization Titer?

In the course of COVID-19 disease, the kinetics of generation and persistence of IgM and IgG antibodies are typically asynchronous and vary with time. In particular, IgM emerges early during primary and secondary immune responses, while IgG typically appears later, but remains in circulation for a longer duration. Akin to IgG, IgM also functions in toxic neutralization, agglutination, complement activation, and acts as a mediator of inflammation. But, since IgM class of antibodies has a shorter persistence in relation to IgG, its detection may be used to indicate a recent event (infection or immunization). In the context of SARS-CoV-2 infection or COVID-19 immunization, determining IgM antibodies early in the event (within 2 weeks that may persist up to nearly 3 weeks following disease onset or immunization) and IgG antibodies (beyond 2 weeks until several months after infection or immunization), have been evidently recognized to be more informative for evaluating antibody-based immunological responses with sufficient sensitivity and specificity (31–33). Interestingly, the beneficial role of IgA, a potent and early SARS-CoV-2–neutralizing agent (34) has been recently documented for intranasal immunization with a Middle East respiratory syndrome coronavirus (MERS)-derived vaccine (35). Viewed in light of these facts, nearly all reports disregard the relevance of IgM despite presenting the data and there is no mention of IgA. Accordingly, future research studies involving a meticulous analysis of the results reflecting on both S-IgM and S-IgA values and establishing a neutralizing titer like that of the conservative IgG (S-RBD) could be helpful to obtain critical complimentary information. In this connection, it could be worth to consider a combined pan-Ig serological tests hat simultaneously measures reactive S-IgM (early antibodies), S-IgA (often detectable early antibodies before IgG), and IgG, like these newly developed assays (33, 36). In the hindsight, the bigger picture of ‘S-pan Ig:neutralization titer’ correlation will perhaps broaden the dataset of serological diagnosis and vaccine assessments regarding our understanding of the early phase of immunological response.

## What Is the Impact of Time Interval Between Prior Infection and Vaccination on Vaccine-Elicited Antibody Responses?

While a slew of studies claim that a single dose of vaccine is sufficient to protect the prior infected individuals as they mounted robust immune response following 1D (1–5), they left a gap in understanding whether the duration of time since resolution of infection had any impact on the level of antibody response following 1D. If ever there is an influence of the former on the latter, would it be different between an asymptomatic cluster and symptomatic subgroup? We have recently passed the anniversary of the first cases of COVID-19 appearing in the U.S., illustrating the point that there is a broad range of time since recovery in the U.S. population. Since protective immune response depends on the level of immunity (immunocompetence), which is a function of time since infection or vaccination, it is also possible that a single dose vaccine may elicit a more robust immune response in a recently recovered individual than in a person recovered more than a year ago or vice versa. In other words, more the period after vaccination (immunity wanes), the greater the susceptibility level to the illness, as reported for other infection scenarios (37, 38). After all, minimizing the susceptible group (waning) is a/the best way to eliminate the infection or epidemic (39). Thus, for a single dose vaccination strategy to be successful in prior-infected group, the feasibility of defining the optimal time interval allowed between prior infection and vaccination, and also determining the clinically reliable threshold of ‘prime-boost’ mechanism (functional immunity/inherent memory of the immune system) must be further explored.

## Conclusions

While clearly endorsing the compelling findings on the adequacy of single-dose vaccination to prior infected cohorts (1–5), we feel detailed analysis and considerations as described here about asymptomatics and waning pools would be an inclusive approach to help define a population (prior-infected) that would benefit from single dose COVID-19 vaccination critically and confidently. Further, as countries prepare to implement novel and customized vaccination programs, addressing these questions in the context of newly emerging variants and breakthrough infections could certainly be impactful, and allowing experts to build upon this idea to enact practices and policies to combat COVID-19. It is appropriate to recall a thorough analysis-based personal view in a recent issue of ‘*The Lancet-Infectious Diseases*’, emphasizing a sustained role for asymptomatic SARS-CoV-2 subset unless the scientific approaches are systematically and accurately approached (40).

## Data Availability Statement

The original contributions presented in the study are included in the article/supplementary material. Further inquiries can be directed to the corresponding author. The code, latest updated estimates, and their visualizations presented in Figure 1 are freely available at a GitHub repository (https://github.com/JungsikNoh/COVID19_Estimated-Size-of-Infectious-Population). 

## Author Contributions

NM, LM, and AM conceived the idea, wrote, and proofread the manuscript. JN provided the data. All authors contributed to the article and approved the submitted version.

## Funding

The data for **Figure 1** was generated from the Lyda Hill Philanthropies-supported, a freely available GitHub repository.

## Conflict of Interest

The authors declare that the research was conducted in the absence of any commercial or financial relationships that could be construed as a potential conflict of interest.

## Publisher’s Note

All claims expressed in this article are solely those of the authors and do not necessarily represent those of their affiliated organizations, or those of the publisher, the editors and the reviewers. Any product that may be evaluated in this article, or claim that may be made by its manufacturer, is not guaranteed or endorsed by the publisher.
